# Gustav Klimt and the Vienna School of Medicine

**DOI:** 10.1007/s00508-025-02503-z

**Published:** 2025-02-21

**Authors:** Markus Müller, Oswald Wagner, Franz Smola

**Affiliations:** 1 Medical University of Vienna, Spitalgasse 23, 1090 Vienna, Austria; 2 Austrian Gallery Belvedere, Prinz Eugen Strasse 27, 1040 Vienna, Austria

## Introduction

**Fig. 1:**
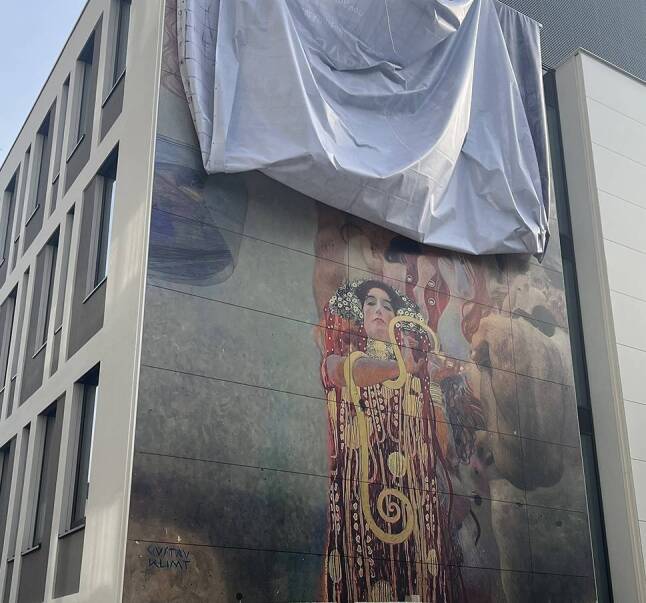
Unveiling of the Façade of the Anna Spiegel Building at the Campus of the Medical University of Vienna with the color reproduction of Klimtʼs faculty painting “Medicine” on 13 November 2024

On 13 November 2024 an approximately 12 × 8 m color reproduction, created using artificial intelligence (AI), of the faculty painting “Medicine” by Gustav Klimt, which was destroyed in 1945, was presented to the public on the facade of a research building at the Allgemeines Krankenhaus (AKH) Wien campus of the Medical University of Vienna (Fig. 1). The AI color reconstruction and realization in the form of a facade was a cooperative effort by a team of the Austrian Gallery Belvedere led by Franz Smola, the company Google Arts and Culture and the manufacturing company Fundermax, St.Veit/Glan, Austria. This very special event for the University also represented a historical arc from the time of the “Second Vienna Medical School” to the present. It also offers an opportunity to take a closer look at Klimt’s relationship with Viennese medicine, which reached a climax in the debate over his faculty paintings.

Like hardly any other artist, Gustav Klimt (1862–1918) represents Austrian modernism and the era “Vienna around 1900” [1–3]. His masterpiece, the painting “Portrait of Adele Bloch-Bauer I”, the so-called Golden Adele, once owned by the Austrian Gallery Belvedere, was restituted to the Altmann family in 2006 [4] and auctioned off as the most expensive painting in the world at that time. It can now be viewed in the Neue Galerie in New York. The dramatic story of this painting has further fuelled the worldwide interest in Klimt, his work, his biography, and the provenance of his artworks [5]. The “Portrait of Adele Bloch-Bauer I” was created during Klimt’s so-called golden period, which lasted from around 1901 to 1909 and was inspired by a visit to the ancient basilicas in Ravenna [6]. Before this period, Klimt worked primarily for public clients, including the “k.k. Ministry of Culture and Education”. Klimt’s commission in 1894 to paint three faculty paintings, including “Medicine”, for the University of Vienna and thereby indirectly for the faculty of the Second Vienna Medical School, led to a legendary art scandal that lasted about a decade at the turn of the century in Vienna. It also represented an important turning point in Klimt’s life and work.

## Klimt and the Second Vienna Medical School

The term Second Vienna Medical School describes the flourishing of enlightened, scientific medicine in Vienna in the nineteenth century and the first decades after the turn of the century. Based on enlightened ideas of the First Vienna Medical School, founded by Gerhard van Swieten in the eighteenth century, the approach of understanding the human organism and its diseases through scientific insight was further pursued. Essential for this era and it’s reasoning were, among other influences, La Mettrie’s reductionist ideas from his work “L’homme machine” [7], Charles Darwin’s theory of evolution, which was repeatedly opposed in Vienna for ideological reasons [8] and the new cell theory that goes back to Antoni van Leeuwenhoek [9]. The pioneers of these modern concepts were in particular the pathologist Carl von Rokitansky and the internist Joseph Skoda [10]. The main tasks of medicine, so they argued, was to accurately describe diseases and perform diagnoses. By applying these new scientific principles, Vienna became a world-renowned center of gravity for medicine. Also, in light of the pessimistic philosophical ideas of Schopenhauer and Nietzsche, therapeutic approaches to change Nature or the natural course of illnesses were viewed as hardly realistic and created the framework for the traditional Viennese “Therapeutic Nihilism”.

By 1888 at the latest, the 26-year-old Klimt came into personal contact with some important representatives of this school. One of his early paintings, which is owned by the Vienna Museum, shows the interior of the old Burgtheater, which was then about to be demolished. It depicts portraits of some of Vienna’s most prominent personalities, such as the surgeon Theodor Billroth and the founder of the world’s first ear nose and throat (ENT) department, Adam Politzer, and his niece Serena Politzer-Lederer, with whom Klimt later developed an intense relationship. Klimt, his brother Ernst and Franz Matsch had already worked for Politzer during their studies at the Vienna School of Applied Arts and had drawn pictures of the auditory system [11, 12]. Klimt also maintained a special friendship with the anatomist Emil Zuckerkandl, whom he met in the salon of his wife Berta Zuckerkandl, daughter of the well-known newspaper publisher Julius Szeps [13]. Klimt was also in contact with Emil’s brothers, Otto Zuckerkandl, a urologist, and Viktor Zuckerkandl. Otto’s wife Amalie and Viktor’s wife Paula were both portrayed by Klimt.

Emil Zuckerkandl, influenced by Rokitansky and Darwin, invited Klimt to take part in dissections to give him an even deeper understanding of the human body and instructed him in embryology and cell theory. Berta Zuckerkandl reports in her memoirs [14]: “*At Klimt’s suggestion, my husband used to give scientific lectures to artists. … Klimt’s palette in particular has been enriched and influenced by this stimulus of the senses.*” These influences can be recognized in several of Klimt’s paintings. For example, in the painting “Danäe” blastocysts are obviously depicted [10, 15], in “Water Snakes I” there are structures reminiscent of sperm cells around an egg, and in several works “fish creatures” are depicted, allegories of Haeckel’s theory that ontogeny follows phylogeny. These developmental biological concepts and influences from philosophical and esthetical ideas of Nietzsche, Schopenhauer and Wagner [3] are reflected in Klimt’s work, for example in repeated depictions of death, birth and pregnancy. Besides, Klimt was also a witness and in the artistic field, a codiscoverer, of the theory of the unconscious. The description of the unconscious and its relationship to sexuality by Sigmund Freud and Arthur Schnitzler is another achievement of the Second Vienna School of Medicine, which Klimt artistically anticipated, foreshadowed and helped develop [3, 10]. In 1918, Klimt died of a stroke in the Vienna General Hospital, the university hospital of the Second Vienna Medical School. Immediately after his death, his young colleague Egon Schiele rushed there to capture the facial features of the dead Klimt in a drawing. Schiele himself would fall victim to the Spanish flu that same year.

In addition to Klimt’s many personal and intellectual connections to the Second Vienna Medical School, Klimt’s probably most intense and, for everyone involved, most unpleasant interaction with the Vienna Medical Faculty was the history of the faculty painting “Medicine”.

## The affair surrounding the faculty painting “Medicine” and its fate

**Fig. 2:**
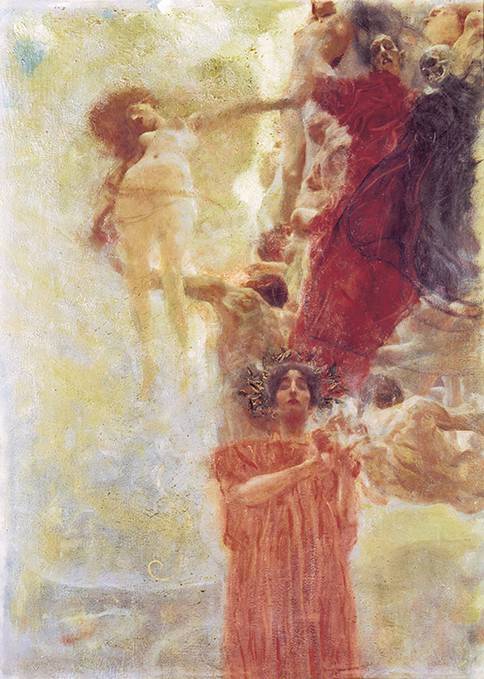
Gustav Klimt, composition sketch for the *Faculty Painting *depicting *Medicine*, 1898, oil on canvas, 72 × 55 cm, Israel Museum, Jerusalem

**Fig. 3:**
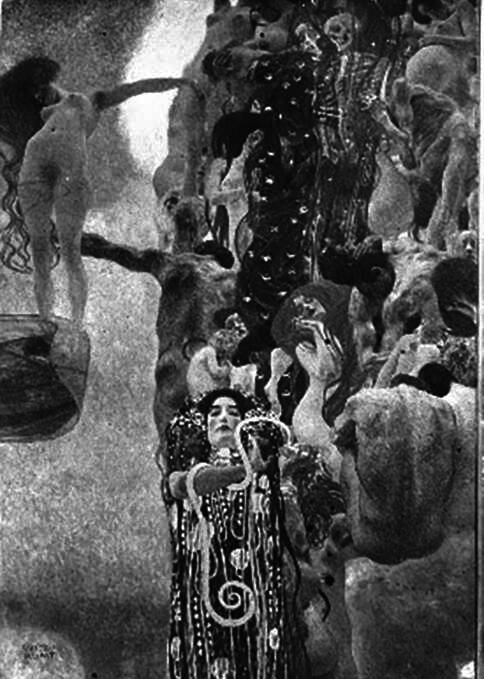
Gustav Klimt, *Faculty Painting:* *Medicine*, 1900–1907, photograph presumably by Moriz Nähr, c. 1900, Austrian National Library, Vienna

In 1894, Gustav Klimt and Franz Matsch received a public commission from the Imperial and Royal k.k. Ministry of Culture and Education to create ceiling paintings for the grand hall of the new building of the University of Vienna, designed by Heinrich von Ferstel and opened on 10 October 1884, on Vienna’s newly built, magnificent boulevard, the *Ringstrasse*. Franz Matsch was supposed to paint “Theology,” Gustav Klimt was supposed to paint “Philosophy,” “Jurisprudence,” and “Medicine.” All four paintings would eventually have a format of 4.3 × 3 m. For this purpose, Klimt made hundreds of sketches, drawings and drafts in the 1890s [16]. Important preliminary studies for “Medicine” are preserved today in the Israel Museum in Jerusalem (Fig. 2), as well as in the Albertina and the Vienna Museum. Likewise, around the turn of the century, several black and white photographs of the different variants of “Medicine” were taken [17, Fig. 3]. A b/w photograph, crucial for the later AI color reconstruction, was taken by Moriz Nähr after 1901 and is now owned by the Austrian National Library.

The presentation of the first faculty painting “Philosophy” in 1900 at the Vienna Secession was followed by heated, polarized public debates. Immediately after the Secession exhibition, the picture was presented at the World Exhibition in Paris, where it won a gold medal. In Vienna, however, it was not to the taste of many academic representatives. According to Schorske, “the traditionalists obviously wanted something similar to Raphael’s School of Athens” [3]. On 24 March 1900, on the initiative of the philosopher Friedrich Jodl, a group of professors at the University, including the dermatologist Ferdinand Hebra and the anatomist Carl Toldt, wrote a petition to Minister of Education Wilhelm von Hartel and spoke out against displaying the painting at the University [18].

In the midst of this dispute over “Philosophy”, the painting “Medicine”, which was not yet fully completed, was presented to the public on 15 March 1901 in the 10th exhibition of the Secession [3, 16, 17, 19, 20]. The presentation of this additional faculty painting further fuelled the debate. Klimt made “not the slightest attempt to present medical science as the doctors thought of it” [3]. Jodl commented in the “Neue Freie Presse” that “the fight is not against naked art nor against free art, but rather against ugly art” [3]. One of Klimt’s opponents was Karl Kraus, who polemicized against Klimt for pages in the popular magazine “Fackel”: “*…Mr. Klimt, who may have realized that we in Vienna need more urgent purchases in the field of medicine than a ceiling painting, in a satirical vein provided his ministerial clients with a picture in which the chaotic confusion of ailing bodies symbolically represents the conditions in the (Vienna) general hospital*.” [21]. The public prosecutor even ordered, unsuccessfully, the confiscation of an issue of the art magazine “Ver Sacrum”, which contained sketches for “Medicine” [3].

Klimt was defended by the art historian Franz Wickhoff, also in a public lecture “What is ugly?”, that was directed against Jodl’s criticism. Alois Riegl, Emil Zuckerkandl, Friedrich Schauta and Hermann Bahr also spoke out in favor of Klimt. In particular, Bahr’s speech “About Klimt” and his collection “Anti Klimt” comprehensively show the arguments of Klimt’s supporters and opponents [22, 23]. There were also intense debates among the University’s staff. The rector, Wilhelm von Neumann, a theologian, was even accused of organizing the protest against Klimt. Berta Zuckerkandl describes this in her memoirs as follows: “*The rector reads out a petition. A protest is to be decided in which the acceptance of the ceiling paintings is described as an insult to art. … Mr. Klimt dares, says one of the deans, to touch what Raphael designed for eternity. He despises the symbols that have been valid in the representation of medicine and philosophy for centuries*.” [14]. A specially appointed “art commission” demanded that “*…* in “Medicine” either a man should be painted instead of the one unclothed female figure or the lady should be given clothing” [24]. In the “Volksblatt” Klimt and Wickhoff were, contrary to fact, equated with “the Jews” [3]. The responsible minister, Hartel, who was personally fond of contemporary art and Klimt, did not give in to public pressure to reject the paintings, but subsequently refused to appoint Klimt as a professor at the art academy.

These years of public debate ultimately led to a change in Klimt’s mood and represent a turning point in his biography. Klimt tried to end the contract with the ministry to keep the pictures. According to the “Neues Wiener Tagblatt” of 11 April 1905, however, “the ministry refused to terminate the contract… Klimt declared that he did not intend to hand over the pictures to the ministry under any conditions and… would only give way to violence” (https://geschichte.univie.ac.at/de/artikel/die-fakultaetsbilder-von-gustav-klimt-im-festsaal-der-universitaet-wien). In a letter to the ministry Klimt emphasized “As is well known, my years of serious effort have brought me numerous insults” [18]. He refused to hand over the paintings and asked to be told where he could deposit the advances he had received (date of receipt: 19 April 1905 [18]). On 27 April 1905, the state accepted to revoke its commission in exchange for repayment of the fee that had already been paid. Klimt, who had apparently faced financial difficulties by this situation, immediately sold “Philosophy” to the Lederer family. A few years later the other two faculty paintings were sold to his fellow painter Kolo Moser. This brought a temporary end to a year-long, heated and public dispute.

**Fig. 4:**
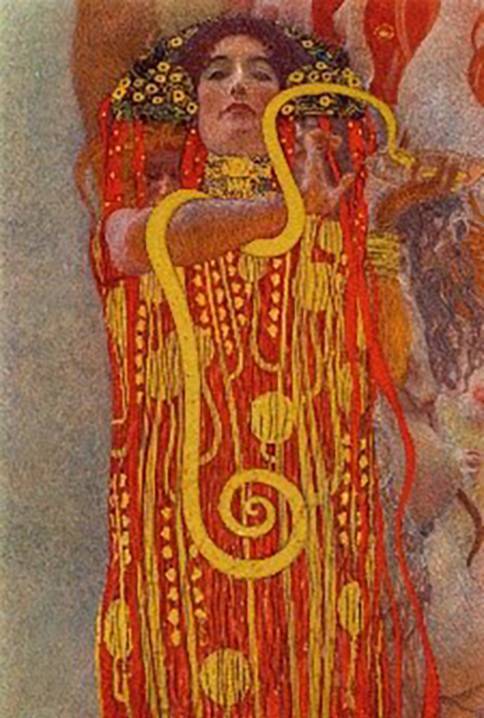
Gustav Klimt, *Faculty Painting:* *Medicine*, 1901, detail with the figure of Hygieia, color reproduction from Max Eisler, *Klimt: Eine Nachlese*, Vienna 1931, plate 10. This is the only color reproduction of the *Faculty Paintings* before their destruction

In 1907 the faculty paintings, including “Medicine,” which had previously been reworked in minor details by Klimt, were now exhibited in their final form in the Miethke Gallery as part of a Klimt solo exhibition. Between 1908 and 1914 Miethke published a folder of collotypes, “The Work of Gustav Klimt,” in which, among other works, “Medicine” was presented in a black and white edition of 300 copies [25, 26]. In 1919 the Austrian Gallery acquired “Medicine” from Kolo Moser’s widow. In 1928, on the 10th anniversary of Klimt’s death, a Klimt memorial exhibition took place, at which the three paintings were shown together again (https://geschichte.univie.ac.at/de/artikel/die-fakultaetsbilder-von-gustav-klimt-im-festsaal-der-universitaet-wien). In 1931, a folder by Eisler, “Gustav Klimt—a gleaning”, was published with a colored part from “Medicine” (Fig. 4), a view of Hygieia, the only colored detail that has ever become known from the original faculty paintings [26].

In 1943, on the initiative of NS “Gauleiter and Reich Governor of Vienna” Baldur von Schirach, a Klimt exhibition took place in the Secession. Schirach, at the suggestion of the Nazi rector and anatomist Eduard Pernkopf, assured that the pictures would be returned to the University; however, they were first taken to a war art depot at Immendorf Castle in Lower Austria to protect them from damage and were irreversibly destroyed there by a fire on 8 May 1945. The Immendorf police report [13] describes the course of events as follows: “*On May 8, 1945 at 6 p. m. a fire broke out in the castle. The SS unit quartered in the castle left the castle on May 8, 1945 at 11 a.m. …. Since the SS unit quartered in the castle was a demolition squad that blew up all the bridges in the area on the same day, it is not unlikely that the fire in the castle was prepared by them as planned and caused by a timed detonator. On May 8, 1945, the Red Army entered Immendorf and drove up to the castle park with 80 to 100 trucks and took up residence in the castle. When the fire broke out at 6 p. m., the Russians quickly left the castle and the park.*” According to Schultheiss, it is “undisputed” that the fire was caused by German soldiers, who emphasized “that it would be a sin if these works of art were seized by Russians hands” [16]. The faculty paintings, which according to Nebehay, were even Klimt’s main work, were therefore lost forever [16]. It was only in 2005 that black and white copies of the faculty paintings were presented on the ceiling of the University’s great hall as part of an exhibition at the Leopold Museum in Vienna.

## Description of the faculty painting “Medicine”

Although Klimt’s interpretations of the faculties are based on traditional allegories, they use an entirely new repertoire of motifs. “Philosophy”, for example, takes the form of a powerful sphinx emerging mysteriously from the fog; “Medicine” is a majestic Hygieia and “Jurisprudence” contains the personifications of *Lex* (law), *Justitia* (justice), and *Veritas* (truth) shown at a practically inaccessible height, while a strange tribunal involving fearsome goddesses of vengeance accompanied by a giant octopus takes place in the foreground. The Faculty Paintings depicting “Philosophy” and “Medicine” also include dense crowds of naked people of different ages and genders, ranging from young children to the aged and from vigorous athletes to pregnant women, crossing the picture space in a seemingly endless vertical stream. In Klimt’s interpretation the people are submissive instruments of dark forces, passive creatures at the mercy of fate. Due to their highly innovative conceptual content and outstanding artistic realization, Klimt’s Faculty Paintings are now considered masterpieces of European Symbolism.

Similar to the first Faculty Painting “Philosophy”, Klimt’s second painting, “Medicine”, had an unusual, split composition. Contrasting a dense stream of people on the right side, the left half of the image features the figure of a woman rising vertically against a bright, luminous sky. She is described as the personification of life, while the personifications of disease and death are clearly depicted in the crowd of people. In the lower half of the image, Hygieia, the goddess of health, holds in her hands a glass bowl from which an Aesculapian snake is drinking. Hygieia is the only figure in the Faculty Paintings for which authentic color information has survived: there is a color illustration of it in the Klimt monograph published by Max Eisler in 1931 ([26, 27], Fig. 4). Additional hints about the colors are provided by a small-format oil sketch of “Medicine”, which Klimt presented at the first meeting of the university commission in 1898 (Fig. 2). Although Klimt modified some areas in the large-format version, the sketch authenticates the general coloring of the final version: the red of Hygieia, the blue of the figure of death, and the brightness of dawn are already present in this small study.

**Fig. 5:**
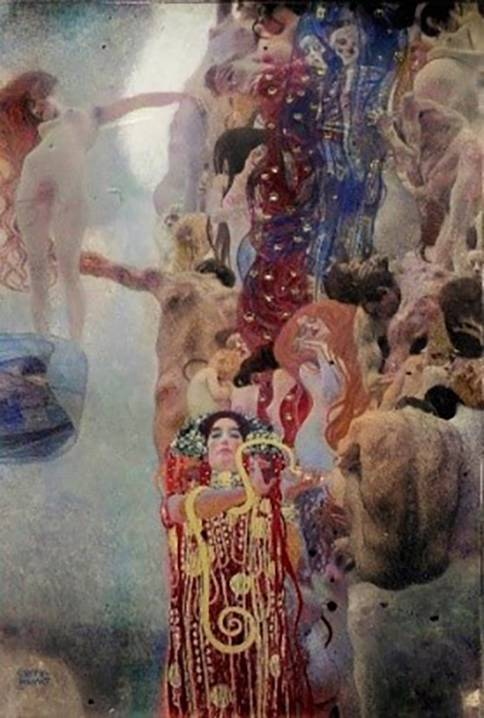
Gustav Klimt, *Faculty Painting: Medicine, 1898–1901*, with minor changes until 1907, oil and gold leaf on canvas. Destroyed in the fire at Schloss Immendorf, Lower Austria, in May 1945. Colorized version from 2021, image by Google/Belvedere

Ludwig Hevesi, Klimt’s eloquent apologist, confirmed and specified these colors in his comments, emphasizing the effect of the powerful red: “While green and blue hues form a cool harmony in the prevailing mood of Philosophy, here [in Medicine], a warmer purple, ranging from rosy to bright and blaring hues, additionally takes effect.” [28]. In another passage, Hevesi even characterizes the painting as “a roaring overture in red.” [29]. Hevesi’s description particularly emphasizes the blue in the figure of death: “In the midst of this harmony full of tenderness and power, however, there is a surprising dissonance. The piercing blue of the veil, which flutters down on the ribs of death. It only wafts through only as a hint, but with all of the sharper, bluer points of the edges and breaks.” [30]. Hevesi also spotted this blue in the veil, which envelops the small child visible in the left half of the image: “And on the other side of the painting, the blue hue resounds again, such an arabesque entwines around the naked body of an infant, who floats off to the side of the swarm.” [30]. Hevesi also finds the clear light of dawn in the sky worthy of mention: “Also the air space […] plays along. It is dawn, the hint of the rising day already reddens the mist, and the first sparks of sunshine want to spray on the horizon.” [30].

## Recreation of the original colors of the faculty painting “Medicine”

While working on the large-scale media project about Gustav Klimt that was initiated by Google Arts & Culture in cooperation with the museum Belvedere in Vienna in 2017 and completed in 2021 [31], the question arose as to whether it would be possible to use artificial intelligence to recreate the original colors of the Faculty Paintings. Searching for suitable experts, Google Arts & Culture found Emil Wallner, an IT specialist from Sweden who had been living in the UK for quite some time. A few years earlier, Wallner had already developed an algorithm that with the help of machine learning is capable of reconstructing the original colors of black and white images. Relying on systems developed by other specialists, he now created a new algorithm to be used specifically for Klimt’s Faculty Paintings. [32].

A key element in Wallner’s new algorithm was the possibility to intervene in this system and to deliver specific color information for selected motifs. Numerous descriptions and accounts penned by art critics and writers in conjunction with the abovementioned controversies when the paintings were first presented fortunately had provided basic knowledge of the color scheme of a large number of motifs in the Faculty Paintings. The reports, many of which are extensive, were helpful in reconstructing the colors of the Faculty Paintings. Concerning the Faculty Painting “Medicine” the abovementioned descriptions made by the art critic Ludwig Hevesi on the occasion of the public presentations of the painting were extremely helpful and quite reliable. On the basis of these text sources alone, it was possible to determine the basic color orientation of more than half of all the motifs appearing in the Faculty Paintings.

All the scholarship-based information on the colors of the Faculty Paintings were inputted into the algorithm. The solutions offered for each motif and detail were then subjected to intense discussion. This led to a detailed dialogue between the programmer and art historian to mutually consider and exchange their perspectives, a process that took place mainly in November and December 2019. The final result achieved several months later can be described as absolutely spectacular (Fig. 5). What were the biggest surprises?

Generally speaking, the intensity of the colors in these monumental works was unexpected. Although the black and white illustrations had always given the impression that the paintings must have been rather dark and pale, the reconstructions now impressively show that the opposite was most likely the case. Due to the abundance of motifs assembled in the paintings, the accumulation of colors increases and leads to a rich palette extending from magically iridescent to festively luminous colors. In the painting “Medicine” the main colors are red combined with blue and radiant white hues. This powerful red and the overall light impression prove to be entirely consistent with the preserved color detail of Hygieia and also confirm to a great extent the effect of the colors, which is already echoed in the surviving early oil sketch. In addition, the differentiation of the colors makes it easier to read the painting as compared with the previous black and white photographs, so that, for example, the positions of the figures in Klimt’s densely packed chains of people are now much easier to understand.

**Fig. 6:**
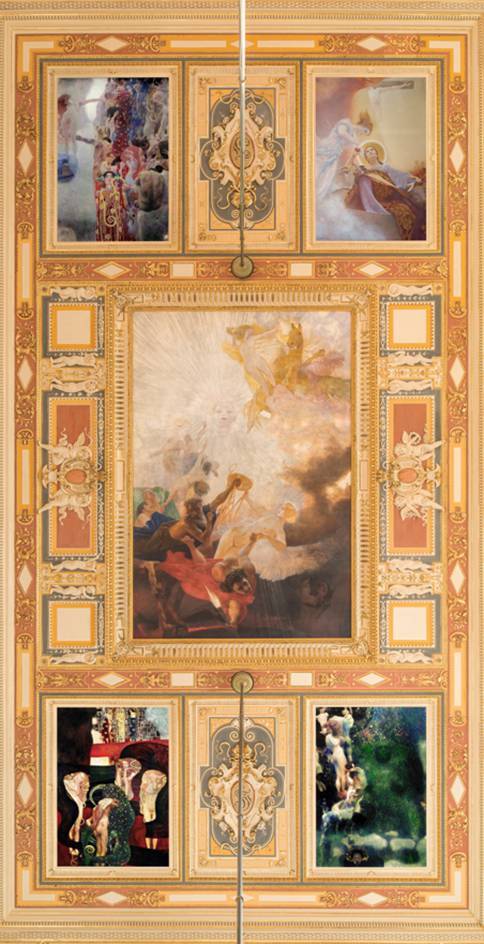
Reconstruction of the planned arrangement of Gustav Klimt’s and Franz Matsch’s *Faculty Paintings* on the ceiling of the Great Hall of the University of Vienna in the color version. Fotocredit: Hannes Buchinger/Montage: checkpointmedia

The reconstruction of the colors of the Faculty Paintings also makes it possible to now suitably appreciate the important role of color in the overall artistic effect of the paintings. After all, it was not least of all the colors, along with the disturbing pictorial narratives and the motifs, that were perceived as provocative, stirring the displeasure and outrage of Klimt’s critics. At the same time, it should not be forgotten that the paintings were meant to be mounted in a hall with a ceiling height of roughly 20 m and accordingly had to be able to convey a special effect even at that distance. Furthermore, it is now even easier to understand how completely different Klimt’s Faculty Paintings were from the two paintings that Franz Matsch had completed for the ceiling of the Great Hall, also in terms of their color schemes, which was one of the commission’s main reasons for not installing Klimt’s works in the Great Hall (Fig. 6).

## Presentation of an AI reconstruction of the faculty painting “Medicine” and Klimt’s references to current art movements in Vienna

In the summer of 2015, the Rector of the Medical University of Vienna, Markus Müller and Vice-Rector Oswald Wagner engaged in discussions about the University’s future. Anticipating a need for more research space, the decision was made to construct new interdisciplinary research buildings. These structures were designed to encourage collaboration across all fields and bridge the gap between preclinical and clinical research. This vision was inspired by Prof. Eugene Braunwald, former Chair of Cardiology at Harvard Medical School, and his insightful keynote lecture at the University’s 10-year anniversary in November 2014. He shared three key messages that resonated deeply: firstly, that precision medicine would bring translational research closer to medical schools; secondly, that medical schools prioritizing translational research alongside basic and clinical research would thrive and thirdly, that strengthening bonds between medical schools and the pharmaceutical industry was crucial. Guided by these principles, we developed a comprehensive plan comprising three new facilities: a Center for Precision Medicine, a Center for Translational Medicine, and a Center for Technology Transfer. The latter was designed to provide space for spin-offs, start-ups and industrial collaborations. In honor of the impact of Prof. Braunwald’s lecture, the new auditorium maximum in the Center for Translational Medicine was named the “*Eugene Braunwald Lecture Hall*”. The Center for Precision Medicine was named “*Eric Kandel Institute*”, recognizing Prof. Kandel’s ongoing and invaluable guidance to our University.

An exciting opportunity arose when a wall of an adjacent research building of the University became available for a mural. After careful consideration Klimt’s reconstructed painting “Medicine” was chosen*,* creating a unique work of art. This idea was conceived in a conversation between Müller and Wagner on a taxi ride to a meeting with Vienna’s Mayor Michael Ludwig in April 2024. Some months later, on 13 November 2024, the AI reconstruction was presented to the public (Fig. 1).

Besides the historical arc of the Second Vienna Medical School to present day medicine, there is also an interesting link between Klimt’s allegories to medicine and contemporary postcolonial art in Vienna. For instance, Gustav Klimt has had a significant influence on the artistic development of the French-Senegalese artist Alexandre Diop, who studied at the Academy of Fine Arts in Vienna. His works, often dissecting the human body into its anatomical components, were recently showcased in the exhibition “Anatomy” in the University’s Museum for the History of Medicine—Josephinum [33]. There, his pieces engaged in a fascinating dialogue with anatomical wax models [33].

Alongside many other western artists, Klimt was fascinated by Africa and African art. In 1897 he painted a portrait of William R. Dowoonah of the Ga from Accra in Ghana [19]. Dowoonah was part of a group of 25 tribe members featured in an ethnographic exhibition at the *Thiergarten am Schüttel* in Vienna as shown on a black and white photo of this group [19]. Klimt later collected African sculptures alongside Asian artworks. Egon Schiele described Klimt’s studio after his last visit: “a square table stood central, surrounded by Japanese woodblock prints and large Chinese paintings. African sculptures were lying on the floor, with a piece of red and black Japanese armor in a corner by the window” [19, 34]. In 1914, Klimt expressed his admiration for Congolese sculptures in a postcard he sent home from Brussels after visiting the Musée du Congo Belge: “They are splendid and magnificent—one feels ashamed that they in their way are so much more capable than we are. I was blown away” [19, 34].

The relationship between African and European art has undergone a remarkable transformation over the past century, evolving from a unidirectional cultural exchange into a rich dialogue of equals. This evolution finds its contemporary expression at the Academy of Fine Arts in Vienna, where artists of African descent exemplify this new paradigm of cultural interchange. At the forefront of this development stand Alexandre Diop and Amoako Boafo, whose parallel journeys add a new chapter to art history. The influence of Viennese Modernism, and particularly Gustav Klimt and Egon Schiele, on these artists manifests distinctively in each artist’s work. Egon Schiele’s eccentric gestural intensity finds new life in their dynamic compositions, while Gustav Klimt’s esthetic sensibilities emerge in unexpected ways. Boafo’s methodical study of Klimt and Schiele reveals his strategic approach to artistic development. His quest to distill the essence of artistic excellence while maintaining authenticity, resulting in a unique portraiture language was showcased in the exhibition “Proper Love” in the Belvedere in 2024 [35], where his paintings were presented side by side with corresponding masterpieces of Gustav Klimt and Egon Schiele. This artistic evolution, taking place in present day Vienna, represents a profound shift in the art world. What began as European appropriation of African artistic elements has transformed into a sophisticated dialogue *en par*, where artists like Boafo and Diop assert creative authority over their cultural heritage.

The worldwide success of these African artists through the lens of Klimt and Viennese Modernism adds historical resonance to their achievements and translates Gustav Klimt’s spectacular concepts to our present time.
